# Parental Social Comparison Related to Tutoring Anxiety, and Guided Approaches to Assisting Their Children's Home Online Learning During the COVID-19 Lockdown

**DOI:** 10.3389/fpsyg.2021.708221

**Published:** 2021-07-30

**Authors:** Qiuyue Yang, Jianjun Gu, Jon-Chao Hong

**Affiliations:** ^1^Department of Education Science, Nanjing Normal University, Nanjing, China; ^2^Institute for Research Excellence in Learning Sciences, Department of Industrial Education, Taiwan Normal University, Taipei, Taiwan

**Keywords:** social comparison, parental tutoring anxiety, guidance strategy, home online learning, COVID-19 pandemic

## Abstract

The COVID-19 pandemic has caused great disruption in education systems around the world. Schools have in some cases ended or limited on-site teaching, and have shifted toward home online teaching. This situation is likely to cause increased uncertainty and anxiety for parents who on one hand may question the quality of home online learning yet, on the other, may not feel sufficiently confident or competent to guide their children's home online learning. Resulting anxiety is expected to be most evident in competitive educational contexts, such as those found throughout much of East Asia. Therefore, using China as the setting, and social comparison theory as the framework, this study examined how variation in parent social comparison relates to parent tutoring anxiety and, in turn, to the types of guided strategies parents use to promote their children's home online learning. The results indicated a positive relationship between parental upward social comparison and parental tutoring anxiety, but a negative relationship between parent downward social comparison and parental anxiety. Parental tutoring anxiety is positively related to the confirmation and structure types, but negatively related to the discovery type of guided approaches. The implication of this study is that parents who resist tendencies of competitive upward social comparison are likely to adopt more effective approaches to guiding their children's home online learning.

## Introduction

At the beginning of 2020, the unprecedented rise of COVID-19 swept China. The National Health Commission announced COVID-19 as a Class B infectious disease as stipulated in the Law of the People's Republic of China on the Prevention and Treatment of Infectious Diseases (People's Republic of China, 2020). To effectively prevent and control the spread of COVID-19, and to ensure the safety and health of teachers and students, the Education Department of Jiangsu Nanjing Province organized home online learning in primary schools beginning on spring semenster, 2020 (Education Department of Jiangsu Province, 2020), forcing teachers and students to adapt to home online learning from February 1, 2020, which then lasted for a year (Murphy, 2020). Many Chinese families invest in private tutoring as part of “intensive parenting.” However, the hiring of tutors may not have been easy during the COVID-19 lockdown. Thus, due to the limited availability of private tutoring at that time, parents in a high educational lever may have taken on the role of providing remedial tutoring for their children themselves (Zhi et al., 2020). The effects of parental tutoring during the COVID-19 lockdown have not yet been extensively studied; thus, the present study focused on exploring parents' perceptions of their individual dispositions related to tutoring affection and cognitive approaches.

Drawing on Bronfenbrenner's (2005) Process-Person-Context (PPC) model, Ashiabi and O'Neal (2015) proposed that school and home are microsystems. In line with the PPC model, parental tutoring anxiety and social comparison can be considered as “person,” the guidance approach as “process,” and home online learning as “context.” In home online learning, parents are an indispensable part of the microsystem which allows Asian children to follow online trajectories. In line with PPC, the guidance strategies created by parents during tutoring may influence their children to successfully tackle tasks (Zhang and Whitebread, 2017). Parental guidance behaviors which have been found to be related to children's learning can be categorized into the three main cognitive aspects of discovery, structuring, and confirmation support (Bevins and Price, 2016). A few studies have shown a parental tendency to adopt multidimensional guidance approaches to explore the contribution to support children's learning; thus, the three types of guidance parents adopted when tutoring their children during the COVID-19 lockdown were explored in this study.

According to social comparison theory (Festinger, 1954), the cognitive model of social anxiety predicts that negative self-focused cognitions increase anxiety when anticipating social comparison (Karasewich and Kuhlmeier, 2020). Parents' social comparison has been defined as a form of psychological control over their children, characterized by parents' behavior of comparing their children's academic achievements to those of other children who are regarded as superior or inferior (Lee et al., 2020), in terms of upward social comparison (USC) or downward social comparison (DSC), respectively. Moreover, tutoring is viewed as a way to cultivate children's academic learning, but at the same time, parents may experience anxiety during the process if they feel that they have insufficient background knowledge (Yamamoto et al., 2016). Researchers have suggested two reasons for Chinese parents' anxiety about their children's home online learning (Bacher-Hicks et al., 2021). First, the limited availability of offline private tutoring during the COVID-19 lockdown may have increased parents' emotional anxiety (Hasan and Bao, 2020). Second, parents may not be familiar with using digital technologies to tutor their young children (Hammer et al., 2020). However, social comparison orientation represents a dispositional tendency to compare oneself to others (Gibbons and Buunk, 1999). During the pandemic, how tutoring anxiety reported by parents was associated with their social comparison orientation was of interest in this study. Therefore, this study examined parental guidance types (GT) within parental tutoring anxiety (PTA) and the link between upward/downward social comparison (USC/DSC) and home online learning during the COVID-19 lockdown.

## Theoretical Background

### Upward and Downward Social Comparison

The theory of social comparison is among the most extensively documented and well-known social psychological conceptions (Chow and Wan, 2017). Social comparison refers to the process of thinking about information about one or more other people in relation to the self (Wood, 1996). Aside from the motives for social comparison, the extensive prior basic research has shown that when engaging in social comparisons, individuals can either compare themselves to someone who is better off than them (upward social comparison, USC) or to someone who is worse off (downward social comparison, DSC; Wood, 1996). In general, such directional comparisons reflect an internal cognitive orientation that may not necessarily be linked to social status (Brown et al., 2007). Accordingly, the present study considered these two types of social comparison that parents may adopt to compare their children's academic performance with that of other children.

Previous studies have suggested that upward and downward social comparisons play a pervasive positive and negative role in our lives (Festinger, 1954; Park and Baek, 2017). Chinese parents' parenting processes and their impact on child development are influenced by deeply embedded Confucian tradition (Fang et al., 2020). For example, compared to their Western counterparts, Chinese parents under the influence of Confucian traditions attach much greater importance to their children's educational development, and like to compare their children's education success as an essential measure of their personal status as well as a central criterion of family prosperity (Ng et al., 2014)). Because few if any studies have explored the tendency of USC and DSC in the context of COVID-19, our study takes on greater importance.

### Parental Tutoring Anxiety

Tutoring is an interactive process by which an experienced adult instructs a child to complete a difficult task that the child finds difficult or cannot complete independently (Wood et al., 1976). Tutoring anxiety refers to a feeling of tension or discomfort when needing to solve learning problems, and is distinct from generalized anxiety and test anxiety (Richardson and Suinn, 1972). It is a subjective emotional state experienced before or during a specific tutoring period related to the act of completing the tutoring itself, the threat of failing, and perceived negative consequences of tutoring (Topping, 2019). Thus, in this study, tutoring anxiety is conceptualized as a situation-specific anxiety experienced by parents who were tutoring their children during the COVID-19 lockdown.

Researchers have estimated that at least 11% of adults experienced severe mathematics anxiety when tutoring children in math (Ching, 2017; DiStefano et al., 2020). With home online learning, the educational responsibilities of parents became even more crucial during the period of the COVID-19 lockdown. Obviously, parents will naturally encounter many difficulties and problems in the process without training and preparation (Prime et al., 2020). Few studies have explored the tutoring anxiety of parents in the context of COVID-19, so this study examined PTA attaching children home online learning.

### Guidance Types

Parental control plays an important cognitive role in children's thoughts and behaviors (Xu et al., 2017). Parental guidance type is a multidimensional concept, including parents' supervision and guidance of children's homework, and providing children with a family structure that contributes to educational success (Yotyodying and Wild, 2014). Although most research examines GT as a whole and integrates different types into a composite index, this approach ignores possible varying effects of different types of guidance strategies (Williams et al., 2020). It is thus advantageous to distinguish the influence of different strategies and to draw more precise conclusions.

Based on how much information and guidance is provided to children, inquiry approaches can take many forms (as shown in Table 1), usually classified based on Herron's (1971) inquiry scale. At the lower end of the continuum, children confirm a phenomenon following specific instructions, with the research question and procedure already provided by the parents, and where the potential results are known in advance (confirmation inquiry). At the upper end, children follow self-directed inquiry by formulating questions, designing procedures and generating explanations based on the evidence collected (open inquiry).

**Table 1:** Types of guided approaches.

**Types of inquiry**	**Guidance**		
	**Question**	**Procedure**	**Results**
Confirmation Type (CT)	+	+	+
Structured Type (ST)	+	+	
Discovery type (DT)	+		
Open			

*+, guidance offered by teachers or parents*.

The last type, the open type, means that parents do not help their children with their homework, procedure, or results. However, few parents in China provide little supervision and assistance when their children have difficulties with their homework (Li et al., 2019). Considering that the samples of this study are Chinese primary school students whose learning methods are not yet mature, homework support is an essential component of guidance types. Therefore, excluding this phenomenon of inaction, we focused on the confirmation, structured, and directed approaches in this study.

### Research Model and Hypotheses

**Figure 1:**
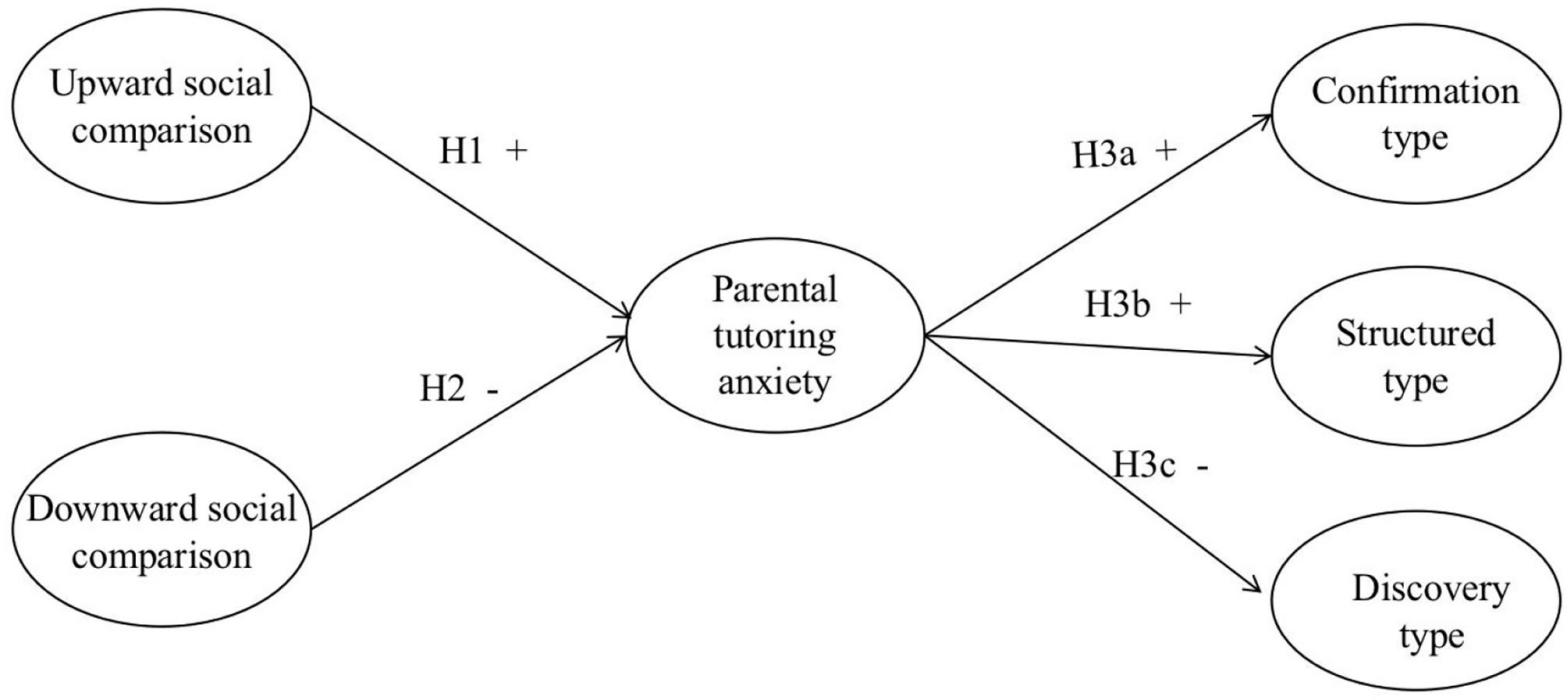
Hypothesized research model.

Drawing on Bronfenbrenner's (2005) Process-Person-Context (PPC) model, and based on the literature review, we constructed a mediation model (shown in Figure 1) to explore the relationships among upward social comparison, downward social comparison, parental tutoring anxiety, and guidance types.

Previous studies (Morry and Sucharyna, 2018) has reported that upward social comparison can influence individuals' mental health and cause more negative emotions, which are risk factors for feelings of stress, anxiety, and inferiority. In turn, there are also many empirical studies (Appel et al., 2016) reporting a significant connection between upward social comparison and parental tutoring anxiety. USC may lead parents to push their children to study harder than others, but this may also contribute to a sense of loss, negative emotions, and increased anxiety if academic outcomes continue to lag compared to those of other children (Wang et al., 2020). Therefore, this study hypothesized that upward social comparison had a significant positive predictive effect on parents' tutoring anxiety, as follows:

H1: USC is positively related to PTA.
  Downward comparison may occur in a variety of ways. Prior research has demonstrated this emotional reactive process by examining how individuals feel after exposure to a description of a social comparison target who is worse off than oneself (Wills, 1981). By contrasting themselves with others who are worse off, individuals may create a lower reference point to evaluate their own situation, causing their own situation to look better than it would have looked otherwise (Robinson et al., 2019). Additionally, those higher in self-centered antagonism report experiencing a greater positive mood after a downward comparison when they were involved in social networking (Burnell et al., 2020). Therefore, this study hypothesized that downward social comparison would have a negative association with parents' tutoring anxiety, as follows:
H2: DSC is negatively related to PTA.
  Parents in general tend to demonstrate a “self-serving” attribution pattern when rating their children's academic competencies, whereby they view their children's abilities as being relatively malleable and impacted by their own influence (Räty and Kasanen, 2010). Goldberg et al. (2021) found that concerned parents tried to provide the best supports possible for their children, experienced worry about adapting to or accepting the reality that their own academic abilities were different from those of their children, and had better performance. In line with this, three hypotheses were proposed regarding how PTA related to the three types of guidance approaches, as follows:
H3a: PTA is positively related to the confirmation type support of children's home online learning.
H3b: PTA is positively related to the structured type support of children's home online learning.
H3c: PTA is positively related to the discovery type support of children's home online learning.
  The importance of learning achievement that the parents attached to the different types of guidance (i.e., guided discovery, guided structuring, and guided performance), for example, have been found to have different effects on promoting children's mathematical reasoning (Baroody et al., 2015), and children's motivation to learn mathematics (Cheung and Kwan, 2021). Particularly, if children encountered a difficult and demanding task, the more likely it was for parents to adopt a confirmation role (Kermani and Brenner, 2000). However, if we combine the three types of guidance to determine the potential for examining the indirect effects in an attempt to identify the mediating aspect of this survey, we may see that the upward and downward social comparisons have specific importance. Thus, how the two types of social comparison related to the three types of parenting guidance mediated by tutoring anxiety was hypothesized as follow.
H4: The two types of social comparison are significantly related to the three types of parenting guidance mediated by PTA.

## Method

### Participants

A total of 496 individuals completed the survey: 237(47.8%) from rural areas and 259 (52.2%) from urban areas of Nanjing in Jiangsu province, China. The effective response rate is 96%. In terms of gender, 80.6% were female; as for age, 6.5% of the parents were 30 years old or less, 51.6% were 31–35 years old, 20.2% were 36–40 years old, 18.5% were 41–45 years old, and 3.2% were 46 years old or above. Most parents had graduated from middle school (51.6%) and high school (22.2%), and most of their children were in grade 3 (42.1%) and grade 2 (36.9%). Participants also reported their gender, education level, political orientation, and annual family income. However, this study only examined the relationships among parental social comparison, parental tutoring anxiety, and parental guidance types, and did not analyze family economic status or other basic information about the parents.

### Procedure

Participants completed an online survey. Snowball sampling was adopted, and a structured questionnaire was presented through an online link. The hyperlink was distributed through WeChat (one of the most popular communication applications in China) beginning on March 15, 2020, and finishing on March 25, 2020 while children were engaged in home online learning. These parents were asked to complete the questionnaire and redistribute it to other parents they knew. In order to ensure the validity of the data, we dropped participants whose response time was below the average response time or who gave invalid answers to all items in the questionnaire.

### Measure

The questionnaire items were adapted from previous studies, and were translated into Chinese. The first step in the adaptation of the instrument was to use the find-and-revise methodology to determine if the instrument did indeed express the same semantic meanings. With the validation of field experts, the instrument was adapted via pilot testing with students to revise the wording. Consequently, the questionnaire included parenting anxiety (8 items), social comparison (upward = 7 items; downward = 7 items), and guidance types (confirmation = 6 items; structured = 6 items; guided = 6 items) in the context of home online learning. All questionnaire items were designed in the form of a 5-point Likert scale (i.e., ranging from 1 indicating strongly disagree to 5 indicating strongly agree); item credibility was subsequently tested.

Social comparison: The present study adopted Brown et al.'s (2007) measure of strategic social comparison motives. This scale provides an index of the extent to which people use social comparison for strategic purposes across a variety of reasons. The base scale consists of items designed to assess strategic social comparison for different purposes, including improvement, valuation, and enhancement. The typical items include*, “I often ask my child to learn from classmates who ‘learn better than him or her’ to evaluate my child's skills”* (evaluation); “*I often ask my child to learn from classmates who ‘learn better than him or her’ to improve my child's ability”* (improvement); “*I often ask my child to learn from classmates who ‘learn better than him or her’ to make my child feel better”* (enhancement).

Parents' tutoring anxiety: Adapted from Dowker et al. (2016), the items measured parents' emotional experience of interacting with children while helping them with their homework. In this study, typical items include, “*When I was helping my child with his/her homework for home online learning, I would feel uneasy if it was too difficult.”*

Guidance types: Herron's (1971) questionnaire items were adapted for this study. Confirmation type (CT): help to find problems or mistakes + help to find ways + help to find answers, for instance: “*When my child has a mistake in his/her math homework, I will let him/her find the mistake to correct first; if he/she can't do it, I will help him/her correct it.”*

Structure type (ST): help to find problems or mistakes + help to find ways + do not help to find answers (open), for example: “*When my child makes a mistake in his/her math homework, I ask him/her to correct it first and then I help him/her correct it.”*

Discovery type (DT): help to find problems or mistakes + do not help to find ways (open) + do not help to find answers (open), for example: “*When my child makes a mistake in his/her math homework, I will directly help him/her correct it.”*

### Data Analysis

The original questionnaire had 37 items in total, including five items for upward social comparison (USC), six for downward social comparison (DSC), eight for PTA, and six each for CT, ST, and DT. After conducting CFA, items with a residual value of more than 0.50 should be deleted (Freund et al., 2010). Hence, 28 items were kept for further analysis, including five items for USC, four for DSC, four for PTA, and five each for CT, ST, and DT. After item reduction, the reliability and validity of the questionnaire constructs were tested.

Composite reliability (CR) and Cronbach's alpha were assessed to evaluate the consistency of the internal model. Moreover, the CR should exceed 0.70, while Cronbach's alpha should be above 0.70 (Fornell and Larcker, 1981). Thus, the constructs reached internal consistency if both CR and Cronbach's alpha were above 0.7. As shown in Table 2, the CR of all constructs was more than 0.70 (ranging from 0.937 to 0.967) and the Cronbach's alpha was also >0.70 (ranging from 0.937 to 0.962). Therefore, the results suggest that the questionnaire had a certain level of reliability and internal consistency of each construct measurement variable.

**Table 2:** Reliability and validity analysis.

**Variables**	***M***	***SD***	**Cronbach's α**	**CR**	**AVE**	**FL**
**threshold**	**–**	**–**	**>0.7**	**>0.7**	**>0.5**	**>0.5**
USC	3.052	0.975	0.947	0.947	0.782	0.884
DSC	2.670	1.025	0.947	0.947	0.818	0.904
PTA	2.809	1.130	0.961	0.962	0.858	0.926
CT	2.605	0.948	0.960	0.960	0.826	0.908
ST	2.909	1.007	0.937	0.937	0.747	0.864
DT	3.229	1.042	0.966	0.967	0.852	0.923

*USC, upward social comparison; DSC, downward social comparison; PTA, parental tutoring anxiety; CT, confirmation type; ST, structured type; DT, discovery type*.

The average variance extracted (AVE) of each construct and the variable measurement condition factor were calculated to access the convergent validity. Freund et al. (2010) suggested that when a construct has sufficient convergent effectiveness, its AVE value should be >0.50. Additionally, when the measurement factor loading of the variable exceeds 0.5, it meets the requirement of convergent validity. As represented in Table 2, the AVE of all constructs was more than 0.50, and the standardized factor loading of each item was >0.50. In sum, the questionnaire had acceptable convergent validity.

In terms of construct discriminant validity analysis (represented in Table 3), normally, the square root of AVE of each dimension must be obtained first, and it must be greater than the absolute value of Pearson correlation coefficient between the two dimensions before each dimension can be expressed as having discriminant validity (Freund et al., 2010). In this study, the square root of the AVE value of all variables is greater than the absolute value of the correlation coefficient between variables, which proves that the measurement model has good discriminative validity.

**Table 3:** Construct discriminative validity analysis.

**Construct**	**USC**	**DSC**	**PTA**	**CT**	**ST**	**DT**
USC	0.884					
DSC	0.049	0.904				
PTA	0.790	0.185	0.926			
CT	0.701	0.130	0.786	0.909		
ST	0.653	0.206	0.724	0.743	0.864	
DT	0.449	0.184	0.404	0.371	0.425	0.923

*USC, upward social comparison; DSC, downward social comparison; PTA, parental tutoring anxiety; CT, confirmation type; ST, structured type; DT, discovery type*.

## Results

### Model Fit Testing

On the basis of Hair et al.'s (2014) recommendation, the measurement model was first measured for acceptable fit to the data by CFA. AMOS 22.0 was used for CFA in order to confirm the validity of the scales, and path analysis was performed to verify the structural relationships among the six factors (Byrne, 2010). In this study, the absolute fit index and relative fit index were used to evaluate the degree of fit of the model. According to Kline (2010), the value of GFI is 0.922 which is more than 0.9 and <1.0. NFI and CFI should both be >0.9. The value of NFI is 0.965 and CFI is 0.986, and the value of RMSEA here is 0.037. From the perspective of the model indexes as Table 4 shows, the χ^2^/*df*, RMSEA, GFI, CFI, NFI, and IFI are all within the acceptable range. Therefore, the model of this study fits well.

**Table 4:** Model fitting analysis.

**Type**	**Fitting index**	**Evaluation standard**	**The fitting results of this model**	**Results**
Absolute fit index	χ^2^/*df*	<3	1.675	Supported
	RMSEA	<0.08	0.037	Supported
	Goodness-of-fit index (GFI)	>0.9	0.922	Supported
	Adjust fitness index (AGFI)	>0.9	0.908	Supported
Relative fit index	Normed fitness index (NFI)	>0.8	0.965	Supported
Incremental fit index	Non-normalized fitness index (NNTI/TFI)	>0.9	0.984	Supported
	Comparative fitness index (CFI)	>0.9	0.986	Supported
	Incremental fitness index (IFI)	>0.9	0.986	Supported
	Relative fitness index (RFI)	>0.8	0.962	Supported
Parsimonious fit index	Streamlining fitness indicators (PGFI)	>0.5	0.783	Supported

### Path Analysis

Through the path analysis of the relationship among variables, the hypotheses of the research model were tested. The analysis results of the path coefficients are shown in Table 5. It can be seen that the significance of the five hypotheses proposed in this study was verified. There are significant states among the hypotheses. All the *p*-values are <0.001. The USC is positively related to PTA (β = 0.834, *t* = 0.043***). The DSC is negatively related to PTA (β = −0.157, *t* = 0.030***). The PTA is positively related to Conformation type (β = 0.830, *t* = 0.031***), and to Structured type (β = 0.782, *t* = 0.036***), but is negatively related to Discovery type (β = −0.440, *t* = 0.040***).

**Table 5:** Path analysis.

**Hypothesis**	**Path**	**Standardized coefficient**	**S.E**.	***p*-value**	**Result**
H1	USC → PTA	0.834	0.043	^***^	Supported
H2	DSC → PTA	−0.157	0.030	^***^	Supported
H3	PTA → CT	0.830	0.031	^***^	Supported
H4	PTA → ST	0.782	0.036	^***^	Supported
H5	PTA → DT	−0.440	0.040	^***^	Supported

*** p < 0.001, **p < 0.01, and *p < 0.05.

USC, upward social comparison; DSC, downward social comparison; PTA, parental tutoring anxiety; CT, confirmation type; ST, structured type; DT, discovery type; *, significant level.

**Figure 2:**
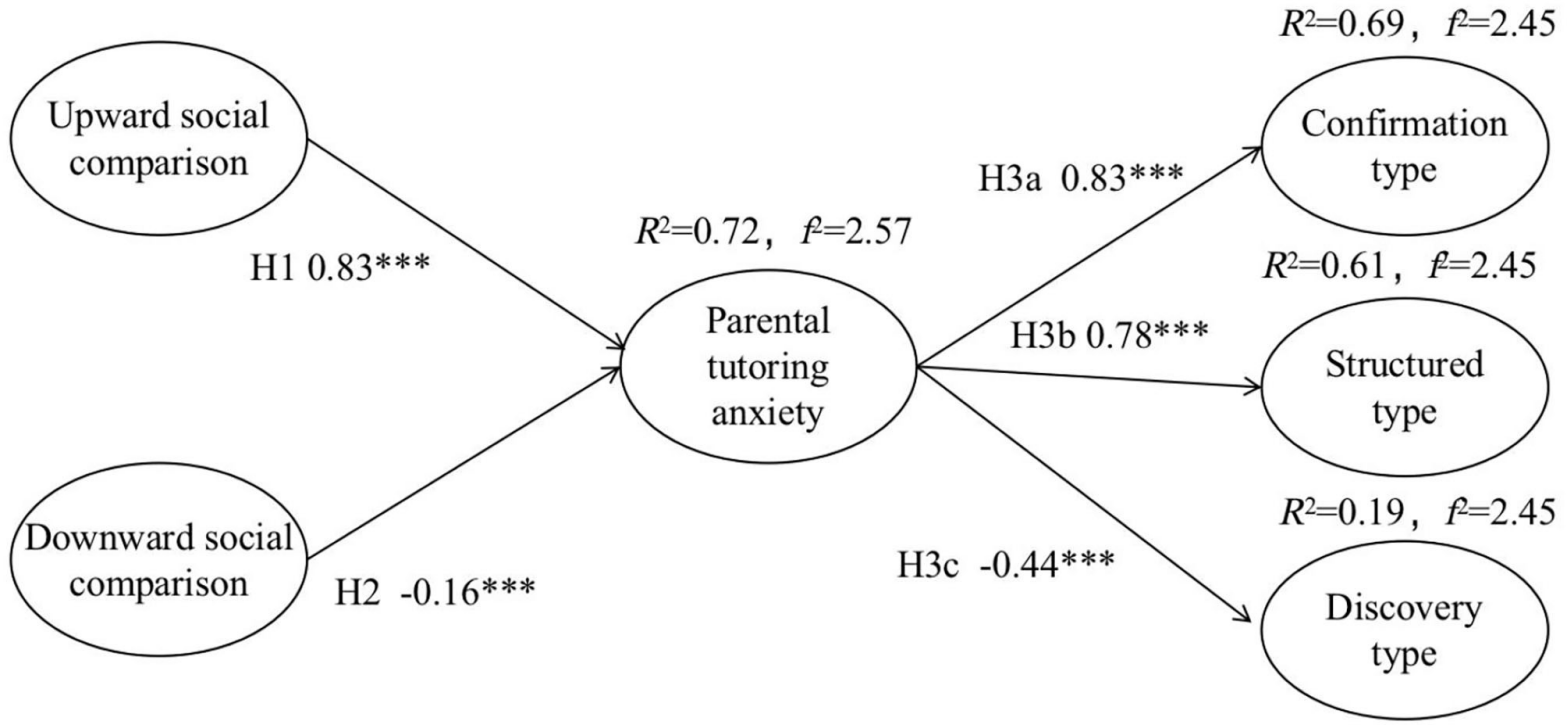
Verified research model. ***p < 0.001, **p < 0.01, and *p < 0.05, *significant level.

The determination coefficient R^2^ quantifies the variance ratio interpreted by the statistical model, and is an important summary statistic of biological benefits. When *R*^2^ values are <0.6, we consider that 0.3–0.6 is medium, and <0.3 is low (Freund et al., 2010). In addition, the effect size of the model (*f*^2^) was proposed by Cohen (1969). This allows researchers to move from a simple recognition of statistical significance to a more general quantifiable description of the size of the effect. *f*^2^ values >0.8 can be considered large. When it is between 0.2 and 0.8, it can be considered medium, and when it is <0.2, it can be considered small. In this study (shown in Figure 2 the explanatory power of USC and DSC on PTA is 72% (*R*^2^ = 0.72, *f*^2^ = 2.57). The explanatory variance of PTA on CT is 69.0% (*R*^2^ = 0.69, *f*^2^ = 2.45). The explanatory variance of PTA on ST is 69.0% (*R*^2^ = 0.69, *f*^2^ = 2.45). The explanatory variance of PTA on DT is 19.0% (*R*^2^ = 0.19, *f*^2^ = 2.45). Hence, the six variables in this study have good predictive power (Hair et al., 2014).

### Indirect Effect Analysis

The results of the mediation analysis are presented in Table 6. The proportions of the total effect of parents' upward and downward social comparison on guidance types attributable to indirect effects were 83%/−16%. The bias-corrected bootstrap 95% CI indicated that USC in the relations between guidance types mediated by PTA were significant (95% CI = [0.393, 0.563]); and the relationship between DSC and guidance types mediated by PTA and were not significant (95% CI = [−0.253, −0.018]), based on Preacher and Kelley's (2011) suggestion. Just as the questionnaire items, parents with upward social comparison, for examples, “*I often compare my children's academic achievements to those of other children who are regarded as superior*,” is positively related to the confirmation type of guided approaches, if they have tutoring anxiety, such as “*If my child makes a mistake in his or her homework, I worry I cannot help him or her correct it directly*.”

**Table 6:** Indirect effects analysis.

**Indirect effects**	**Effect of value**	**SE**	**Bias-corrected 95%CI**		***p***
			**Lower**	**Upper**	
USC → PTA → GT	0.4738	0.0428	0.3929	0.5631	<0.001^***^
DSC → PTA → GT	−0.1682	0.0429	−0.2526	−0.0181	>0.05

*USC, upward social comparison; DSC, downward social comparison; PTA, parental tutoring anxiety; GT, guidance types;*

*** p < 0.001, *signicant level.

## Discussion

Guided by social comparison theory (Stefano et al., 2020), the present study expanded on previous research by simultaneously examining the effects of upward social comparison and downward social comparison on guidance types mediated by parents' tutoring anxiety in a sample of Chinese primary school students' parents. This study demonstrated that parents' upward social comparison is a crucial predictor of parents' tutoring anxiety, while downward social comparison reduces their anxiety. Moreover, the present research extends the prior literature by showing that parental tutoring anxiety has an indirect effect on the association between social comparison and the different types of guidance.

The prediction of USC to emotional anxiety is supported by a previous study (Park and Baek, 2017). The present study found that the relationship between parents' USC and tutoring anxiety was positive. This may be explained by the fact that more than 40 years of reform and opening up in China have not only brought rapid economic development, but have also led to increasingly serious social polarization (Samek et al., 2020). Moreover, during the COVID-19 period, the high potential of private tutoring may increase parents' anxiety by creating unequal access to online learning resources among families with different socioeconomic backgrounds (Lopez-Agudo et al., 2020). In such circumstances, parents are more likely to be prone to anxiety over the possibility of becoming losers in comparison with others (Zhang et al., 2020). In line with this, H1 was positively supported.

According to the self-worth theory (Adams et al., 2017), people with low self-worth are relatively likely to also exhibit low-effort and low-avoidance tendencies (Stefano et al., 2020). Additionally, those higher in self-centered antagonism report experiencing greater positive emotion after a downward comparison when they are involved in social networking (Burnell et al., 2020). Therefore, despite great social pressure toward competition, parents who experience an optimistic and relaxed attitude with their children have a relatively low level of tutoring anxiety (Beckerman et al., 2017). Furthermore, the present findings also support the notion that parental DSC is negatively associated with tutoring anxiety.

Parents with different levels of social status showed significant differences in how they rated the importance of their children's learning performance (Cheung and Kwan, 2021). The importance of learning achievement that the parents attached to the different types of guidance (i.e., guided discovery, guided structuring, and guided performance), for example, has been found to have different effects on promoting children's mathematical reasoning (Baroody et al., 2015), and children's motivation to learn mathematics (Cheung and Kwan, 2021). Concerned parents try to provide the best possible guidance they can for their children, and they experience worry about adapting to or accepting the reality that their own academic abilities are different from those of their children, and expecting their children have better academic performance than them (Goldberg et al., 2021). In examining H3a, H3b, and H3c, the results indicated that PTA was positively related to the confirmation and structuring types of guidance, but was negatively related to the discovery type of guidance. This result reveals that parents with lower levels of tutoring anxiety adopted the discovery approach to guide their children to hone their online learning. Moreover, parents with higher levels of tutoring anxiety were significantly more likely to choose the confirmation and structuring types of guidance.

Particularly, if children encounter a difficult and demanding task, the more likely it is for parents to adopt a directive role (Kermani and Brenner, 2000). However, if we combine the three types of guidance to ascertain the potential for examining the indirect effects, we may see the importance of upward and downward social comparisons related to guidance type in our attempt to unveil the mediating effects of PTA. In examining H5, the present study showed that the indirect effect of tutoring anxiety between parental USC and guidance types was positive, but no significance existed between DSC and guidance types.

Similar to prior research of other countries, American parents are experiencing particularly marked anxiety during the pandemic, due not only to pandemic-related uncertainties, but also to tutoring with children who are home online learning (Freisthler et al., 2021; Hong et al., 2021). It has also been found that parents tend to make upward comparisons about their children's academic achievements is closely related to parental anxiety (Lee et al., 2020) Especially in Korea, known for its strong collectivistic culture, parents tend to compare their children to their superior peers, producing a sense of anxiety (Guimond et al., 2006). In consistence to the above studies, our findings indicated that an association of parental involvement in the homework process with parental social comparison mediated by parental anxiety during COVID-19.

## Conclusion

Drawing on the PPC model, the study explored the correlates between parents' social comparison, tutoring anxiety, and guidance approaches. The results contribute to the literature in the following key ways. First, we used a dataset from China to explore the potential effects of parental guidance types, which is of major importance to educational policy in China and also has implications for the Asia-Pacific region as well as globally during the COVID-19 pandemic lockdown. Second, this study is the first to highlight parental tutoring anxiety as a specific indicator to evaluate guidance types and examine the indirect effects of subjective factors between social comparison and parents' guidance types.

### Implications

As one of the first explorations of parental tutoring anxiety and guidance types using social comparison theory during COVID-19, this study provides important information to guide children involved in home online learning. The findings suggest that Chinese parents need to decrease their USC so as to guide their children's home online learning, in line with reducing their parenting anxiety. Second, being sensitively aware of parental difficulties due to the technology usage that parents encounter and that affect their tutoring of their children's online learning, workshops on using remote learning systems may be needed for those parents.

Third, the ecological theory considers the embeddedness of children's development in learning content at home through processing online systems (Claudia and Marcela, 2020). Schools need to communicate with parents to find out what kind of supplementary materials they need to support parents' tutoring of their children's home online learning. Finally, the guided approach leverages children's existing knowledge and skills to find and internalize new knowledge and problem solving (Bevins and Price, 2016). As the discovery type of guidance was predicted by a low level of PTA in this study, parents can reduce their tutoring anxiety by considering downward social comparison.

### Limitations and Future Studies

Several limitations of this study should be noted. Most notably, the survey was conducted during the early stage of home online learning in Jiangsu, Nanjing province, China. A wider sampling of socioeconomic status levels and other geographical locations is recommended for the purposes of confirming the validity and applicability of these findings. Second, this study was largely comprised of elementary school children's parents. Admittedly, previous researchers have shown that parents adjust their guidance types in accordance with their children's age and level of competence (Silinskas and Kikas, 2019); thus, future studies may include different school age groups in order to make comparisons with the constructs in this study. Third, this study only measured parental tutoring anxiety and how they guided their children's homework, but did not capture other important aspects (e.g., the characteristics of children's learning and their reaction to their parents' different guidance types); future research is needed to investigate these issues more thoroughly. Finally, compared to American parents, Chinese parents tend to use more content instructions to instruct their children (Hou et al., 2020), indicating that cultural issues affect guidance types. Future studies may compare parents' social comparison tendency, tutoring anxiety levels, and guidance types based on parents living in different cultural contexts.

## Data Availability Statement

The original contributions presented in the study are included in the article/supplementary material, further inquiries can be directed to the corresponding author/s.

## Author Contributions

All authors listed have made a substantial, direct and intellectual contribution to the work, and approved it for publication.

## Conflict of Interest

The authors declare that the research was conducted in the absence of any commercial or financial relationships that could be construed as a potential conflict of interest.

## Publisher's Note

All claims expressed in this article are solely those of the authors and do not necessarily represent those of their affiliated organizations, or those of the publisher, the editors and the reviewers. Any product that may be evaluated in this article, or claim that may be made by its manufacturer, is not guaranteed or endorsed by the publisher.

## References

[B1] Adams K. E. Tyler J. M. Calogero R. Lee J. (2017). Exploring the relationship between appearance-contingent self-worth and self-esteem: the roles of self-objectification and appearance anxiety. Body Image 23:176. 10.1016/j.bodyim.2017.10.004 29055772

[B2] Appel H. Gerlach A. L. Crusius J. (2016). The interplay between facebook use, social comparison, envy, and depression. Curr. Opin. Psychol. 9, 44–49. 10.1016/j.copsyc.2015.10.006

[B3] Ashiabi G. S. O'Neal K. K. (2015). Child social development in context: an examination of some propositions in Bronfenbrenner's bioecological theory. Sage Open. 5, 24–45. 10.1177/2158244015590840

[B4] Bacher-Hicks A. Goodman J. Mulhern C. (2021). Inequality in household adaptation to schooling shocks: COVID-induced online learning engagement in real time. J. Public Econ. 193, 34–39. 10.1016/j.jpubeco.2020.104345 PMC8486492 34629567

[B5] Baroody A. J. Purpura D. J. Eiland M. D. Reid E. E. (2015). The impact of highly and minimally guided discovery instruction on promoting the learning of reasoning strategies for basic add-1 and doubles combinations. Early Child. Res. Q. 30, 93–105. 10.1016/j.ecresq.2014.09.003

[B6] Beckerman M. Van Berkel S. R. Mesman J. Alink L. R. A. (2017). The role of negative parental attributions in the associations between daily stressors, maltreatment history, and harsh and abusive discipline. Child Abuse Neglect 64, 109–116. 10.1016/j.chiabu.2016.12.015 28081496

[B7] Bevins S. Price G. (2016). Reconceptualising inquiry in science education. Int. J. Sci. Educ. 38, 1–13. 10.1080/09500693.2015.1124300

[B8] Bronfenbrenner U. (2005). The bioecological theory of human development, in Making Human Beings Human: Bioecological Perspectives on Human Development, ed Bronfenbrenner U. (Thousand Oaks, CA: Sage), 3–15.

[B9] Brown D. J. Ferris D. L. Heller D. Keeping L. M. (2007). Antecedents and consequences of the frequency of upward and downward social comparisons at work. Org. Behav. Hum. Decis. Proc. 102, 59–75. 10.1016/j.obhdp.2006.10.003

[B10] Burnell K. Ackerman R. A. Meter D. J. Ehrenreich S. E. Underwood M. K. (2020). Self-absorbed and socially (network) engaged: Narcissistic traits and social networking site use. J. Res. Pers. 84:103898. 10.1016/j.jrp.2019.103898 32863468 PMC7451760

[B11] Byrne B. M. (2010). Structural equation modeling using AMOS, in Basic Concepts, Applications, and Programming, ed Byrne B. M. (New York, NY: Routledge Press), 29–48.

[B12] Cheung S. K. Kwan J. L. Y. (2021). Parents' perceived goals for early mathematics learning and their relations with children's motivation to learn mathematics. Early Child. Res. Q. 56, 90–102. 10.1016/j.ecresq.2021.03.003

[B13] Ching B. H. H. (2017). Mathematics anxiety and working memory: longitudinal associations with mathematical performance in Chinese children. Contemp. Educ. Psychol. 51, 99–113. 10.1016/j.cedpsych.2017.06.006

[B14] Chow T. S. Wan H. Y. (2017). Is there any Facebook depression'? Exploring the moderating roles of neuroticism, Facebook social comparison and envy. Pers. Individ. Diff. 119, 277–282. 10.1016/j.paid.2017.07.032

[B15] Claudia M. A. Marcela P. (2020). Home alone versus after-school programs: the effects of adult supervision on child academic outcomes. Int. J. Educ. Res. 104, 145–163. 10.1016/j.ijer.2020.101601

[B16] Cohen J. (1969). Statistical power analysis for the behavioural sciences. New York: Academic Press, 35–49.

[B17] DiStefano M. O'Brien B. Storozuk A. Ramirez G. Maloney E. (2020). Exploring math anxious parents' emotional experience surrounding math homework-help. Int. J. Educ. Res. 99, 23–30. 10.1016/j.ijer.2019.101526

[B18] Dowker A. Sarkar A. Looi C. Y. (2016). Mathematics anxiety: what have we learned in 60 years? Front. Psychol. 7:e508. 10.3389/fpsyg0.2016.00508 27199789 PMC4842756

[B19] Education Department of Jiangsu Province (2020). Further Work Will Be Coronavirus Pneumonia Epidemic Prevention and Control Work[N]. Available online at: http://www.moe.gov.cn/jyb_xwfb/xw_zt/moe_357/jyzt_2020n/2020_zt03/zydt/zydt_dfdt/202001/t20200129_416854.html (accessed January 29, 2020).

[B20] Fang S. Huang J. Wu S. Jin M. Kim Y. Henrichsen C.. (2020). Family assets, parental expectation, and child educational achievement in china: a validation of mediation analyses. Child. Youth Serv. Rev. 112, 53–57. 10.1016/j.childyouth.2020.104875

[B21] Festinger L. (1954). A theory of social comparison processes. Hum. Relat. 7, 117–140. 10.1177/001872675400700202

[B22] Fornell C. Larcker D. F. (1981). Structural equation models with unobservable variables and measurement error: algebra and statistics. J. Mark. Res., 18, 382–388. 10.1177/002224378101800313

[B23] Freisthler B. Gruenewald P. J. Tebben E. McCarthy K. S. Price J. (2021). Understanding at-the-moment stress for parents during COVID-19 stay-at-home restrictions. Soc. Sci. Med. 279, 1–8. 10.1016/j.socscimed.2021.114025 34004571 PMC9756775

[B24] Freund R. J. Wilson W. J. Mohr D. L. (2010). Data and Statistics. New York, NY: Springer Press, 221–241.

[B25] Gibbons F. X. Buunk B. P. (1999). Individual differences in social comparison: development of a scale of social comparison orientation. J. Pers. Soc. Psychol. 76, 129–142. 10.1037/0022-3514.76.1.129 9972558

[B26] Goldberg A. E. McCormick N. Frost R. Moyer A. (2021). Reconciling realities, adapting expectations, and reframing “success”: adoptive parents respond to their children's academic interests, challenges, and achievement. Child. Youth Serv. Rev. 120:105790. 10.1016/j.childyouth.2020.105790

[B27] Guimond S. Chatard A. Martinot D. Crisp R. J. Redersdorff S. (2006). Social comparison, self-stereotyping, and gender differences in self-construals. J. Pers. Soc. Psychol. 90:221. 10.1037/0022-3514.90.2.221 16536648

[B28] Hair J. F. Black W. C. Babin B. J. Anderson R. E. (2014). Multivariate Data Analysis, 7th Edn. Upper Saddle River, NJ: Pearson Prentice Hall.

[B29] Hammer M. Scheiter K. Stürmer K. (2020). New technology, new role of parents: How parents' beliefs and behavior affect students' digital media self-efficacy. Comput. Hum. Behav. 116, 34–38. 10.1016/j.chb.2020.106642

[B30] Hasan N. Bao Y. (2020). Impact of “e-learning crack-up” perception on psychological distress among college students during covid-19 pandemic: a mediating role of “fear of academic year loss”. Child. Youth Serv. Rev. 118, 21–28. 10.1016/j.childyouth.2020.105355 32834276 PMC7422835

[B31] Herron M. D. (1971). The nature of scientific enquiry as seen by selected philosophers, science teachers and recent curricular materials. Sch. Rev. 79, 171–212. 10.1086/442968

[B32] Hong J. Y. Choi S. Cheatham G. A. (2021). Parental stress of Korean immigrants in the U.S.: meeting child and youth's educational needs amid the COVID-19 pandemic. Child. Youth Serv. Rev. 127:106070. 10.1016/j.childyouth.2021.106070 PMC8554711 34728872

[B33] Hou S. Wang R. Liu Y. (2020). How parental instructions scaffold young children's learning performance: a cross-cultural comparison between America and China. Cogn. Dev. 56:100953. 10.1016/j.cogdev.2020.100953

[B34] Karasewich T. A. Kuhlmeier V. A. (2020). Trait social anxiety as a conditional adaptation: a developmental and evolutionary framework. Dev. Rev. 52:100886. 10.1016/j.dr.2019.100886

[B35] Kermani H. Brenner M. E. (2000). Maternal scaffolding in the child's zone of proximal development across tasks: cross-cultural perspectives. J. Res. Child. Educ. 15, 30–52. 10.1080/02568540009594774

[B36] Kline R. B. (2010). Principles and Practice of Structural Equation Modeling. New York, NY: The Guilford Press, 29–51.

[B37] Lee Y. Ha J. H. Jue J. (2020). Structural equation modeling and the effect of perceived academic inferiority, socially prescribed perfectionism, and parents'forced social comparison on adolescents' depression and aggression. Child. Youth Serv. Rev. 108:104649. 10.1016/j.childyouth.2019.104649

[B38] Li Y. Hu T. T. Ge T. S. Auden E. (2019). The relationship between home-based parental involvement, parental educational expectation and academic performance of middle school students in mainland china: a mediation analysis of cognitive ability. Int. J. Educ. Res. 97, 139–153. 10.1016/j.ijer.2019.08.003

[B39] Lopez-Agudo L. A. Marcenaro-Gutiérrez O. D. Molina-Marfil J. A. (2020). School tutoring and academic performance: a too close relationship? Stud. Educ. Eval. 66, 21–24. 10.1016/j.stueduc.2020.100903

[B40] Morry M. M. Sucharyna T. A. (2018). Relationship social comparisons in dating and marital relationships: adding relationship social comparison interpretations. J. Soc. Psychol. 89, 1–19. 10.1080/00224545.2018.1498826 30058945

[B41] Murphy M. P. A. (2020). COVID-19 and emergency e-learning: consequences of the securitization of higher education for post-pandemic pedagogy. Contemp. Secur. Policy 41, 1–14. 10.1080/13523260.2020.1761749

[B42] Ng F. F. Y. Pomerantz E. M. Deng C. (2014). Why are Chinese mothers more con-trolling than American mothers? “My child is my report card.” Child Dev. 85, 355–369. 10.1111/cdev.12102 23581633 PMC3714336

[B43] Park S. Y. Baek Y. M. (2017). Two faces of social comparison on Facebook: the interplay between social comparison orientation, emotions, and psychological well-being. Comput. Hum. Behav. 79, 83–93. 10.1016/j.chb.2017.10.028

[B44] People's Republic of China (2020). National Health Council Bulletin 2020, No. 1[N]. Available online at: http://www.gov.cn/xinwen/2020-01/21/content_5471158.htm (accessed January 29, 2020).

[B45] Preacher K. J. Kelley K. (2011). Effect size measures for mediation models: quantitative strategies for communicating indirect effects. Psychol. Methods 16, 93–115. 10.1037/a0022658 21500915

[B46] Prime H. Wade M. Browne D. T. (2020). Risk and resilience in family well-being during the COVID-19 pandemic. Am. Psychol. 75, 631–643. 10.1037/amp0000660 32437181

[B47] Räty H. Kasanen K. (2010). A seven-year follow-up study on parents' expectations of their children's further education. J. Appl. Soc. Psychol. 40, 2711–2735. 10.1111/j.1559-1816.2010.00677.x

[B48] Richardson F. C. Suinn R. M. (1972). The mathematics anxiety rating scale: psychometric data. J. Counsel. Psychol. 19, 551–554. 10.1037/h003345612674278

[B49] Robinson A. Bonnette A. Howard K. Ceballos N. Dailey S. Lu Y.. (2019). Social comparison, social media addiction, and social interaction: an examination of specific social media behaviors related to major depressive disorder in a millennial population. J. Appl. Biobehav. Res. 24, 1–14. 10.1111/jabr.12158

[B50] Samek A. Cowell J. M. Cappelen A. W. Cheng Y. Decety J. (2020). The development of social comparisons and sharing behavior across 12 countries. J. Exp. Child Psychol. 192, 34–41. 10.1016/j.jecp.2019.104778 31958667 PMC11309852

[B51] Silinskas G. Kikas E. (2019). Math homework: Parental help and children's academic outcomes. Contemp. Educ. Psychol. 59, 67–69. 10.1016/j.cedpsych.2019.101784

[B52] Stefano R. Sonia I. Rubinia C. Bonfantib G. Lo C. (2020). The role of online social comparison as a protective factor for psychological wellbeing: a longitudinal study during the COVID-19 quarantine. Pers. Individ. Diff. 4, 34–41. 10.1016/j.paid.2020.110486 33169042 PMC7610095

[B53] Topping K. J. (2019). A theoretical model of intergenerational tutoring. J. Intergener. Relat. 18, 88–105. 10.1080/15350770.2019.1646182

[B54] Wang W. Wang M. Hu Q. Wang P. Jiang S. (2020). Upward social comparison on mobile social media and depression: the mediating role of envy and the moderating role of marital quality. J. Affect. Disord. 270, 22–28. 10.1016/j.jad.2020.03.173 32339106

[B55] Williams K. E. So K. T. Siu T. S. C. Lindsey D. (2020). A randomized controlled trial of the effects of parental involvement in supported playgroup on parenting stress and toddler social-communicative behavior. Child. Youth Serv. Rev. 118, 56–62. 10.1016/j.childyouth.2020.105364

[B56] Wills T. A. (1981). Downward comparison principles in social psychology. Psychol. Bull. 90, 245–271. 10.1037/0033-2909.90.2.245

[B57] Wood D. Bruner J. Ross G. (1976). The role of tutoring in problem solving. J. Child Psychol. Psychiatry 17, 89–100. 932126 10.1111/j.1469-7610.1976.tb00381.x

[B58] Wood J. V. (1996). What is social comparison and how should we study it? Pers. Soc. Psychol. Bull. 22, 520–537. 10.1177/0146167296225009

[B59] Xu J. Z. Fan X. T. Du J. X. He M. Y. (2017). A study of the validity and reliability of the parental homework support scale. Measurement 95, 93–98. 10.1016/j.measurement.2016.09.045

[B60] Yamamoto Y. Holloway S. D. Suzuki S. (2016). Parental engagement in children's education: motivating factors in Japan and the U.S. Sch. Commun. J. 26, 45–66.

[B61] Yotyodying S. Wild E. (2014). Antecedents of different qualities of home-based parental involvement: findings from a cross-cultural study in Germany and Thailand. Learn. Cult. Soc. Interact. 3, 98–110. 10.1016/j.lcsi.2014.02.002

[B62] Zhang F. Jiang Y. Ming H. Ren Y. Wang L. Huang S. (2020). Family socio-economic status and children's academic achievement: the different roles of parental academic involvement and subjective social mobility. Br. J. Educ. Psychol. 90, 34–40. 10.1111/bjep.12374 32777088

[B63] Zhang H. Whitebread D. (2017). Linking parental scaffolding with self-regulated learning in Chinese kindergarten children. Learn. Instruct. 49, 121–130. 10.1016/j.learninstruc.2017.01.001

[B64] Zhi K. Chen Y. Huang J. (2020). Children's self-control and family savings for education: an empirical examination from China. Child. Youth Serv. Rev. 119, 75–81. 10.1016/j.childyouth.2020.105575

